# Application of Environmental Scanning Electron Microscope-Nanomanipulation System on Spheroplast Yeast Cells Surface Observation

**DOI:** 10.1155/2017/8393578

**Published:** 2017-04-27

**Authors:** Maryam Alsadat Rad, Mohd Ridzuan Ahmad, Masahiro Nakajima, Seiji Kojima, Michio Homma, Toshio Fukuda

**Affiliations:** ^1^Department of Control and Mechatronic Engineering, Faculty of Electrical Engineering, Universiti Teknologi Malaysia, 81310 Skudai, Johor, Malaysia; ^2^Department of Micro-Nano Systems Engineering, Nagoya University, Nagoya, Japan; ^3^Division of Biological Science, Graduate School of Science, Nagoya University, Nagoya, Japan

## Abstract

The preparation and observations of spheroplast W303 cells are described with Environmental Scanning Electron Microscope (ESEM). The spheroplasting conversion was successfully confirmed qualitatively, by the evaluation of the morphological change between the normal W303 cells and the spheroplast W303 cells, and quantitatively, by determining the spheroplast conversion percentage based on the OD_800_ absorbance data. From the optical microscope observations as expected, the normal cells had an oval shape whereas spheroplast cells resemble a spherical shape. This was also confirmed under four different mediums, that is, yeast peptone-dextrose (YPD), sterile water, sorbitol-EDTA-sodium citrate buffer (SCE), and sorbitol-Tris-Hcl-CaCl_2_ (CaS). It was also observed that the SCE and CaS mediums had a higher number of spheroplast cells as compared to the YPD and sterile water mediums. The OD_800_ absorbance data also showed that the whole W303 cells were fully converted to the spheroplast cells after about 15 minutes. The observations of the normal and the spheroplast W303 cells were then performed under an environmental scanning electron microscope (ESEM). The normal cells showed a smooth cell surface whereas the spheroplast cells had a bleb-like surface after the loss of its integrity when removing the cell wall.

## 1. Introduction

Over the years, traditional scanning electron microscope (SEM) made a lot of contributions in the imaging of materials with a detailed description of their structure and surfaces [1–4]. However, the need for high vacuum operating environment and complex sample preparation steps made the application on biological samples and specimen unfavorable [5–7]. Environmental Scanning Electron Microscope (ESEM) has overcome many of the drawbacks found in SEM and introduced a new advantage in biological research [8–11]. The ability of this tool to operate in wet and gaseous atmospheres made the nonconductive samples conductive, thus overcoming the need for coating samples prior to characterization and preserving samples original features for further testing and manipulation [12–14]. In addition, the capability to control water vapor pressure inside the microscope while operating keeps the samples hydrated, which increases the chances of survival [15, 16].

One of the earliest experiments on biological specimens was done by Collins et al. [17]. They explained the application and advantages of using ESEM on microorganisms. ESEM has found its potentials in tissue engineering and biomaterials studies, because it supports the observation of cell and their topography in hydrated atmospheres [18]. Furthermore, the reduced sample preparation steps are useful for investigating mammalian cells and biomaterial interactions [5, 19–23]. Kirk et al. have imaged mammalian cells using ESEM showing very fine detail of delicate features such as filopodia and membrane ruffles [24]. They also showed that the cells survived the initial stages of sample preparation but experienced some damage during the dehydration stage. They have suggested cells with stronger cell wall could be imaged in their living states.

**Figure 1:**
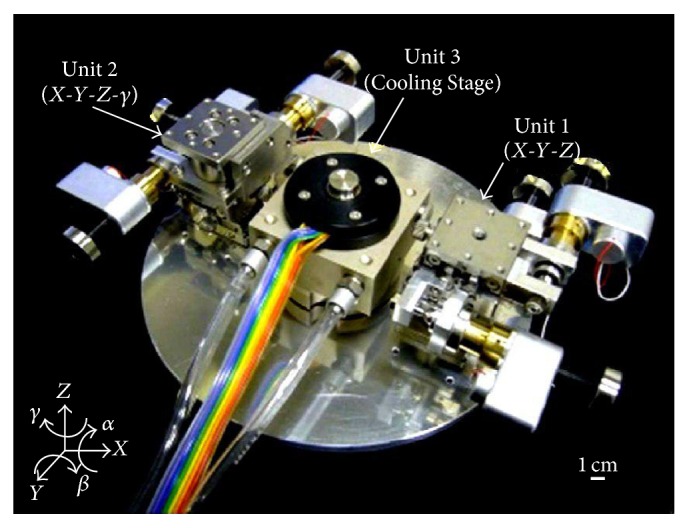
Nanorobotic manipulator of ESEM.

One of the main criteria of ESEM is the real life observation and monitoring of biological cells. This could be an advantage by integrating tools such as nanomanipulators or patch clamp to analyze cells and their characteristics while performing experiments. From our previous work, a nanomanipulator was successfully incorporated inside ESEM (Figure 1) for various single cell manipulation and characterization [25, 26].

One potential application of ESEM is to study the ion channel current measurement of cells by combining it with a patch-clamp system. The conventional planar patch-clamp system requires a bath solution to perform the measurement. However, this could result in having come errors in the measurement because of the differences between the bath solution chemical properties and the cytoplasm inside the cell [27]. To overcome that, instead of using the bath solution, the electrodes could be injected into the cell and the cytoplasm could be used as the medium for the current to flow during the current recording. Spheroplasting is one of the early steps required in the ion channel measurements experiments [28]. Thus, in order to perform the patch-clamp experiment inside ESEM, it is important to confirm the ability of ESEM to observe spheroplast cells successfully before further manipulation could be carried out.

Nanomanipulation is an effective strategy for the characterization of basic properties of individual nanoscale objects and to construct nanoscale devices quickly and effectively. We have constructed a hybrid nanorobotic manipulation system integrated with a transmission electron microscope- (TEM-) nanorobotic manipulator (TEM manipulator) and a scanning electron microscope- (SEM-) nanorobotic manipulator (SEM manipulator) [29]. This system allows effective sample preparation inside SEM with wide working area and many degrees of freedom (DOFs) of manipulation. It has high resolution measurement and evaluation of samples inside a TEM capability. The sample chambers of these electron microscopes are set under the high vacuum (HV) condition to reduce the disturbance of electron beam for observation. To observe the water-containing samples, for example, biocells, drying treatment processes are additionally needed. Hence, direct observations of water-containing samples are normally quite difficult in these electron microscopes.

In the present study, we used the nanorobotic manipulators inside an ESEM [30]. It has been constructed with 3 units and 7 degrees of freedom (DOFs) in total (Figure 1). The ESEM enables direct observation of water-containing samples with nanometer high resolution by a specially built secondly electron detector. The evaporation of water is controlled by both the sample temperature (0–40°C) and sample chamber pressure (10−2600 Pa). The temperature of the sample is controlled by the cooling stage unit (Unit 3). The detailed specifications of the manipulator and the ESEM can be obtained from our previous paper [31]. The following experiments have been conducted through this system. The observation and comparison of W303 cells and spheroplast W303 were successfully performed using ESEM. Spheroplast W303 cells were obtained by enzymatic digestion which were confirmed qualitatively by the comparison of cell morphology between the cells and spheroplast cells, and quantitatively, by the OD_800_ absorbance data. The successful observation of spheroplast cells opens the possibility for a new way in the single ion channel current measurement.

## 2. Materials and Methods 

### 2.1. Cell Cultures

Wild type yeast cells (W303 strains) were used for the observations and measurements under optical microscope and ESEM system. The W303 cells were cultured on a YPD plate (1% yeast extract, 2% peptone, 2% glucose, and 2% agar) in a 37°C incubator for 48 hours. A single colony was then picked from the cultured plate and then dipped into a tube containing 10 mL of YPD media. The tube was then incubated overnight in 30°C at 200 rpm. The OD600 values of the samples were measured by a spectrophotometer and samples that had OD600 values between 0.2 and 0.3 were used for spheroplasting.

### 2.2. Preparation of W303 Spheroplasts

Spheroplasts were prepared using Pichia spheroplast kit. In brief, logarithmic growing W303 wild type yeast cells (OD_600_ value between 0.2 and 0.3 in 1 mL of culture) were harvested by centrifugation at 6000 rpm for 5 minutes at 30°C and then washed with 1 mL of sterile water. Cells were pelleted by centrifugation at 6000 rpm for 5 minutes at 30°C.

The cell pellets were washed by resuspending in 1 mL of SED buffer containing 1 M sorbitol, 25 mM EDTA pH 8.0, and 1 M dithiothreitol and then centrifuged at 6000 rpm for 5 minutes at 30°C. The cells then were washed with 1 mL of 1 M sorbitol and centrifuged at 6000 rpm for 5 minutes at 30°C. Then, they were resuspended by swirling in 1 mL of SCE medium. A 3 *μ*L of cell wall hydrolyzing enzyme, Zymolyase, was added to the cells. The cells were then incubated at 30°C for about 1 hour. The spheroplasts were washed with 1 mL of 1 M sorbitol and collected by centrifugation at 6000 rpm for 5 minutes at 30°C. They were then resuspended in 1 mL of CaS.

**Figure 2:**
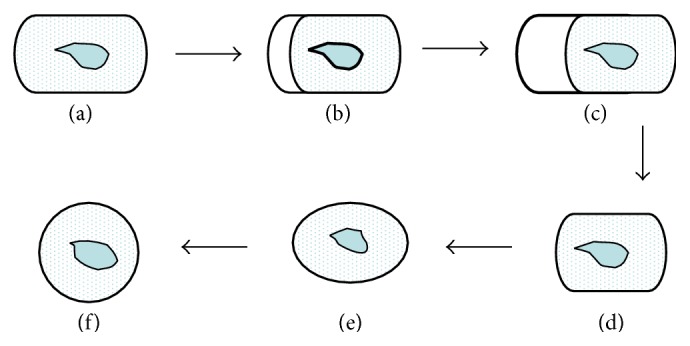
Schematic diagram illustration of the two phases in spheroplast formation. (a)–(d), Cell turns into prospheroplast. The appearance of spheroplast can be seen when the prospheroplast changes into a spherical shape ((e), (f)).

A schematic diagram illustrating the spheroplast conversion is presented in Figure 2. In the first phase, the W303 cells were transformed into prospheroplasts. The prospheroplasts were extruded at one end where partial digestion of the cell wall occurred (Figures 2(a)–2(d)), retaining its shape despite the apparent loss of a supporting wall. In the second phase, the prospheroplasts rapidly transformed into spheroplasts where a spherical shape became dominant (Figures 2(e) and 2(f)).

### 2.3. W303 Spheroplast Observation by an Optical Microscope

The spheroplast cells were observed in four different mediums: YPD, sterile water, SCE, and CaS using Olympus IX-71 optical microscope at room temperature (20–25°C) in 100× oil immersion objective lens. The initial 1 mL spheroplast cells inside CaS medium were aliquoted into four microcentrifuge tubes. The first aliquot was the spheroplasts inside CaS medium. The other three aliquots were centrifuged at 6000 rpm for 5 minutes in 30°C and the CaS supernatant was discarded completely. The spheroplast pellets inside each of the three tubes were then resuspended with 250 *μ*L of YPD medium, 250 *μ*L of sterile water, and 250 *μ*L of SCE medium, respectively.

### 2.4. W303 Spheroplasts Observations by ESEM

The ESEM system can perform direct observation of water-containing samples with nanometer high resolution by specially built secondary electron detector. The evaporation of water is controlled by adjusting the sample's temperature (~0–~40°C) and sample's chamber pressure (10–2600 Pa). The temperature of the sample is controlled by the cooling stage unit, that is, Unit3, as shown in Figure 1. ESEM system has a capability to control the chamber's pressure from high vacuum (~10^−4^ Pa) to high humidity (10–2600 Pa). The detailed specifications of the manipulator and the ESEM can be obtained from our previous paper [26]. The nanomanipulator has a tungsten probe, which has been used to transfer the single cell using the adhesion force. This force is produced between the micro probe and cell. In fact, the nanomanipulator system can control the position of a single cell.

## 3. Results and Discussion

### 3.1. Cells Morphology under Optical Microscope

**Figure 3:**
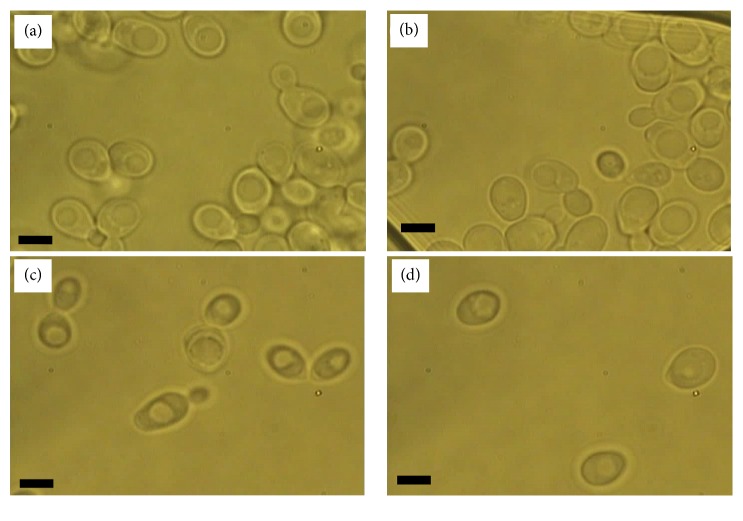
The morphology of the W303 cells inside four different mediums: (a) YPD, (b) sterile water, (c) SCE, and (d) CaS. Bar scale is 5 *μ*m.

**Figure 4:**
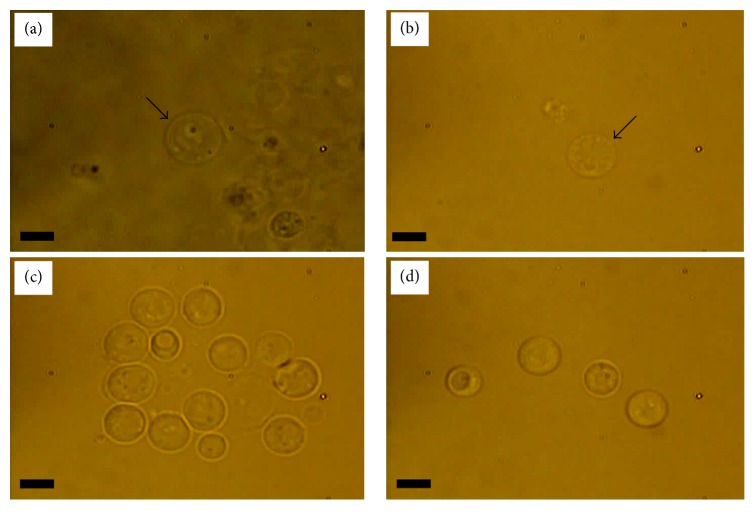
The morphology of the spheroplast W303 cells inside four different mediums: (a) YPD, (b) sterile water, (c) SCE, and (d) CaS. Arrow marks are added to indicate the position of the spheroplast cells inside the first two mediums, that is, YPD and sterile water. Bar scale is 5 *μ*m.

Figures 3 and 4 show a comparison between the morphology of W303 cells before and after spheroplasting inside four different mediums: YPD, sterile water, SCE, and CaS. From the observations, the morphology of the W303 cells inside all four mediums had an oval shape.

YPD medium provided the best living medium for the cells, followed by sterile water, SCE, and CaS mediums (Figures 3(a)–3(d)). The reduction in number of the W303 cells in SCE and CaS media compared to the YPD (1% yeast extract, 2% peptone, 2% glucose, and 2% agar) is due to the absence of yeast extract. The yeast extract will typically contain all the amino acids necessary for growth.  Figure 4 shows the morphology of spheroplast cells inside four the four mediums. It is clear that the spheroplast cells displayed a spherical shape inside all mediums. However, the condition and visibility of cells inside YPD and sterile water mediums were poor as compared to the SCE and CaS medium (Figures 4(a)–4(d)). The spherical shape for the spheroplast yeast cells was also reported by [32].

### 3.2. Spheroplast Conversion Percentage Based on the OD_800_ Absorbance Data

The percentage of the spheroplast cells conversion can be determined from the following equation:

(1) $$\begin{array}{c} \%\;\text{Spheroplast}=100-\left[\;\left(\;\frac{\left({\left.{OD}_{800}\right|}_{\text{time}=t}\right)}{\left({\left.{OD}_{800}\right|}_{\text{time}=0}\right)}\;\right)\times 100\;\right]. \end{array}$$

**Figure 5:**
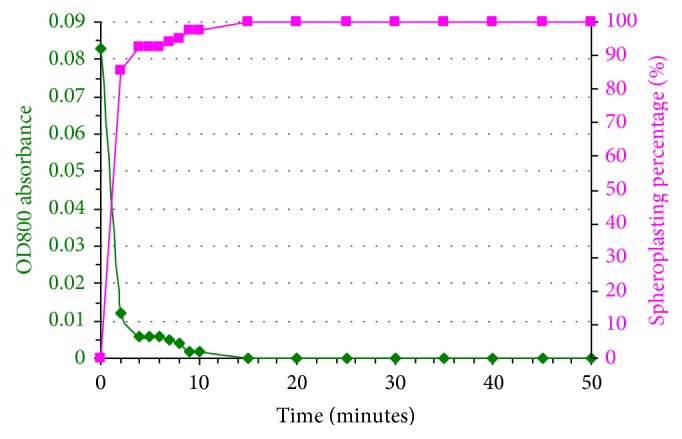
The OD_800_ absorbance data and its corresponding spheroplast conversion percentage for 50 minutes of treatment using digestion enzyme; Zymolyase, on W303 cells.

Figure 5 shows the values of OD_800_ absorbance data for 50 minutes and its corresponding spheroplast conversion percentage. From the graph, it is shown that the cells rapidly changed into spheroplast cells; 85% of the cells converted into spheroplasts in 2 minutes after the addition of the digestion enzyme. The whole W303 cells completely adapted the spheroplast cells conditions in 15 minutes. From this data, it is concluded that the spheroplasting experiment was successfully achieved.

### 3.3. Surface Characteristic of W303 Cells and Spheroplast W303 Cells under ESEM

**Figure 6:**
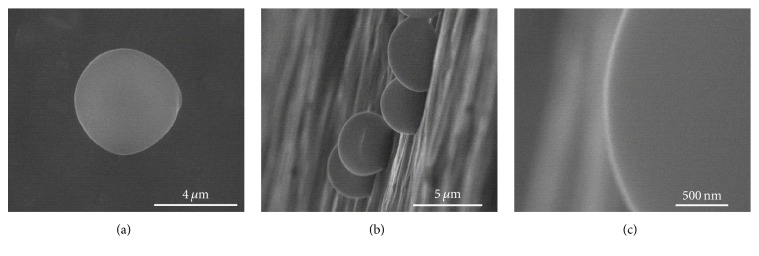
The surface topology of the W303 cells under an ESEM from different views: (a) top view, (b) side view, and (c) closed-up side view.

Figure 6 shows the surface topology of the cells under ESEM. The observations were performed at 30 kV and 100 *μ*A. The environmental parameters settings were 600 Pa and 0°C. The whole cells were kept inside the YPD medium and diluted with distilled water. From the observation, the surfaces of the W303 cells were very smooth as can be seen (top view (Figure 6(a)), side view (Figure 6(b)), and closed-up side view (Figure 6(c))). This is also confirmed in previous literature [25, 26].

**Figure 7:**
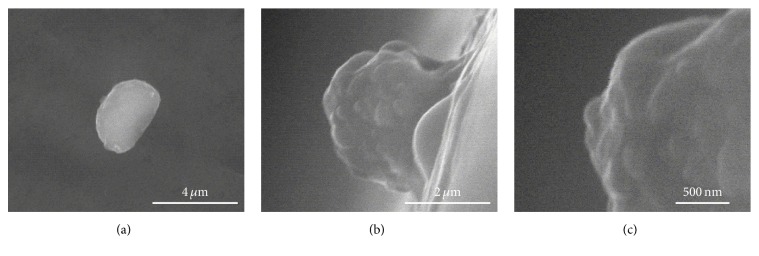
The surface topology of the spheroplast W303 cells under an ESEM from different views: (a) top view, (b) side view, and (c) closed-up side view.

Figure 7 represents the surface topology of the spheroplast cells under an ESEM. For the first time, the observations of spheroplast cells were performed without the need of complex sample preparation and sample coating. This will ensure the viability of the spheroplast cells that will enable further characterization and analysis to be carried out. As expected, the surface topology of the spheroplast cells was not smooth as cells having a cell wall. The significance of the cell wall as a cell shaper was highly acknowledged from these observations. In addition to the nonsmooth surface topology, the spheroplast cells experienced some reduction in size as can be seen in top view image of spheroplast cell (Figure 7(a)). From the side view images (Figures 7(b) and 7(c)), it is noticed that the surface had several bleb structures.

## 4. Conclusion 

The advantages of the integrated ESEM-nanomanipulation system rely on its capability to perform in situ local direct observation and manipulation of biological sample and the ability to control the environmental conditions. The observations of the spheroplast without prior coating for an electron microcopy observation have been highlighted in this work. To the best of our knowledge, this work is the first to attempt to observe spheroplast cells under electron microscope without the need of sample coating. The spheroplasting was verified qualitatively and quantitatively by observing the cell's morphological change under optical microscope observations and the spheroplast conversion percentage based on the OD_800_ absorbance data. The spheroplast cells showed a spherical shape as compared to the oval shape for the normal cells. The electron microscope observation revealed the bleb-like surface of the spheroplasts as compared to the very smooth surface of the normal cells. This work could be extended to perform single ion channel current measurements on the spheroplast W303 cells inside the ESEM-nanomanipulation system.
